# An educational intervention to update health workers about HIV and infant feeding

**DOI:** 10.1111/mcn.12922

**Published:** 2019-12-17

**Authors:** Christiane Horwood, Lyn Haskins, Ameena Goga, Tanya Doherty, Vaughn John, Ingunn M.S. Engebretsen, Ute Feucht, Nigel Rollins, Max Kroon, David Sanders, Thorkild Tylleskar

**Affiliations:** ^1^ Centre for Rural Health University of KwaZulu‐Natal Durban South Africa; ^2^ Health Systems Research Unit South African Medical Research Council Cape Town South Africa; ^3^ Department of Paediatrics University of Pretoria Pretoria South Africa; ^4^ School of Public Health University of the Western Cape Cape Town South Africa; ^5^ School of Education University of KwaZulu‐Natal Pietermaritzburg South Africa; ^6^ Centre for International Health University of Bergen Bergen Norway; ^7^ Research Centre for Maternal, Fetal, Newborn and Child Health Care Strategies University of Pretoria Pretoria South Africa; ^8^ Maternal and Infant Health Care Strategies Research Unit South African Medical Research Council Cape Town South Africa; ^9^ Department of Maternal, Newborn, Child and Adolescent Health World Health Organization Geneva Switzerland; ^10^ Department of Neonatology, Faculty of Health Sciences University of Cape Town and Mowbray Maternity Hospital Cape Town South Africa; ^11^ Department of Paediatrics and Child Health University of Cape Town Cape Town South Africa

**Keywords:** breastfeeding, clinical practice guideline, health worker, HIV, infant feeding, primary health care, South Africa

## Abstract

Clinical guidelines are used to translate research findings into evidence‐based clinical practice but are frequently not comprehensively adopted by health workers (HWs). HIV and infant feeding guidelines were revised by the World Health Organization to align feeding advice for HIV‐exposed and unexposed infants, and these were adopted in South Africa in 2017. We describe an innovative, team‐based, mentoring programme developed to update HWs on these guidelines. The intervention was underpinned by strong theoretical frameworks and aimed to improve HWs' attitudes, knowledge, confidence, and skills about breastfeeding in the context of HIV. On‐site workshops and clinical mentoring used interactive participatory methods and a simple low‐tech approach, guided by participants' self‐reported knowledge gaps. Workshops were conducted at 24 participating clinics over three sessions, each lasting 1–2 hr. Evaluation data were collected using a self‐administered questionnaire. Of 303 participating HWs, 249/303 (82.2%) attended all workshops. Achieving high workshop attendance was challenging and “catch‐up” sessions were required to achieve good coverage. Common knowledge gaps identified included antiretroviral therapy adherence monitoring during breastfeeding and management of viral load results (173 participants), management of breast conditions (79), and advice about expressing and storing breastmilk (64). Most participants reported all their knowledge gaps were addressed and anticipated that their practice would change.

We describe a feasible, sustainable approach to updating HWs on HIV and infant feeding guidelines and improving skills in breastfeeding counselling in resource‐constrained settings. This approach could be adapted to other topics and, with further evaluation, implemented at scale using existing resources.

**Key messages**

- Provision of health education and support for breastfeeding are critical health worker skills, however, frequent changes to infant feeding guidelines for HIV exposed infants has led to confusion among health workers and reduced the skills, knowledge, and confidence to support breastfeeding in high HIV prevalence areas like South Africa.
- Revised WHO guidelines for HIV and infant feeding align feeding recommendations for HIV‐exposed infants with those for all infants, but evidence is lacking about effective approaches that can be used to update health workers in low–middle‐income countries on new clinical guidelines.
- We present an on‐site, team‐based mentoring approach for updating clinic‐based health workers on new HIV and infant feeding guidelines, which was based on strong theoretical educational frameworks and successfully implemented using a simple local‐tech participatory methodology aimed at improving attitudes and beliefs of health workers as well as their knowledge and skills.

## 1 INTRODUCTION

Clinical guidelines are an established way of translating evidence‐based medicine into good quality, evidence‐based clinical practice. Health workers (HWs) are expected to provide care according to a variety of guidelines and, in addition, are expected to adapt their practice when these guidelines change. Translating new evidence into clinical practice is an active process involving not just changes to HWs' knowledge but also to their attitudes and self‐efficacy, and whether new guidelines are adopted depends on factors related to both the health system and to individual HWs. Providing information or training alone does not result in HWs implementing new knowledge and skills, a more systematic approach is required to achieve this (Haines, Kuruvilla, & Borchert, 2004). Challenges of applying new research evidence through adapted or revised guidelines are compounded in resource‐constrained settings, where infrastructure is poor, HW shortages, and rapid turnover of staff are the norms (Sodhi et al., 2014). In addition, ability to change practices may be beyond the control of individual HWs (Jansson, Syrjälä, Talman, Meriläinen, & Ala‐Kokko, 2018).

Continuous medical education (CME) to maintain and support HWs knowledge and skills is not provided systematically for front‐line HWs in most health systems in resource‐constrained settings. The authors' experience is that CME is usually conducted as ad hoc, project‐funded, out‐of‐clinic workshops for a small number of HWs with the intention that the new knowledge will trickle down to fellow HWs, which rarely succeeds. Interventions are often short term and may be driven by external funding with inadequate provision for sustainability (Egger et al., 2017). A wide variety of different approaches can be used for CME, either singly or in combination, including circulating guidelines, sending reminders, interactive workshops, didactic teaching, clinical audit and feedback, in‐service training, and quality improvement plan–do–study–act cycles (Althabe et al., 2008; Ganz et al., 2013). Effect sizes are usually small and there is limited evidence of effectiveness of such interventions in resource‐constrained settings and of their scalability, particularly in rural areas (Althabe et al., 2008).

The importance of translating knowledge into clinical practice for maternal and child health to address the know–do gap is well recognised (Blank et al., 2013; Kebede et al., 2014; Siron, Dagenais, & Ridde, 2015), but many life‐saving maternal and child health interventions are not fully implemented leading to lost opportunities for preventing morbidity and mortality (Boerma et al., 2018). Strategies shown to be effective in changing clinical practice are those that involve tailoring core messages for the local context, as well as the needs, level of understanding, and demands of the end user (Siron et al., 2015). Messages should be packaged in a way that is user‐friendly, familiar to the target audience, relevant, and understandable (Poot et al., 2018). It is also important to evaluate and address barriers to implementation (Vermond et al., 2018). However, closing the know‐do gap continues to be a major challenge to achieving high coverage of evidence‐based interventions (Kebede et al., 2014).

Building on the World Health Organization (WHO) 2010 HIV and infant feeding guidelines (WHO & United Nations Children Fund, 2010), updated 2016 WHO guidelines aligned infant feeding advice for HIV‐exposed and unexposed infants. These guidelines recommend breastfeeding for up to 24 months for women who are antiretroviral therapy (ART) adherent and virally suppressed (WHO & United Nations Childrens Fund, 2016). In South Africa, these guidelines were adapted by the National Department of Health with the Circular Minute No. 3 of 2017/2018 HIV/AIDS, TB & MCWH and an amendment of the 2013 Infant and Young Child Feeding policy, which was circulated to provincial and district managers. This elicited a need to update South African HWs about the change in guidelines.

Counselling HIV‐infected pregnant and breastfeeding mothers is an area of prime importance in Southern Africa due to the high HIV prevalence among pregnant women and provides an example of challenges faced by front‐line HWs when recommended practices change. In the past decade, frequent and often substantial changes to the HIV and infant feeding guidelines have resulted in the promotion of changing and, at times, contradictory infant feeding messages, many of which were not supportive of breastfeeding (Chinkonde, Hem, & Sundby, 2012). This has led to confusion among HWs and mothers receiving mixed and inadequate messages (Doherty et al., 2019; Horwood, Jama, Haskins, Coutsoudis, & Spies, 2019; Kafulafula, Hutchinson, Gennaro, & Guttmacher, 2014). In addition to these frequent changes, providing appropriate feeding counselling is a complex and demanding task with HWs being asked to assess and manage individual risk on the basis of the woman's adherence to ART, viral load monitoring, general child health issues, and breastfeeding skills. The attitudes and personal experience of HWs, as well as their relevant knowledge and skills, have an impact on decision making about HIV management, particularly about provision of infant feeding advice and support. HWs are also members of the communities where they work, and HIV is a complex social issue that impacts on all community members in high prevalence settings (Nieuwoudt & Manderson, 2018). As a result, the context in which infant feeding counselling occurs for HIV‐infected women is influenced by multiple factors (Schuster, McMahon, & Young, 2016).

To bridge a gap in disseminating the new HIV and infant feeding guidelines, we designed and implemented an innovative, team‐based, participatory mentorship intervention. This intervention was underpinned by a strong theoretical framework and educational principles and aimed to address the attitudes and beliefs of HWs about breastfeeding in the context of HIV, in addition to improving their knowledge, skills, and confidence. The intervention was based on the premise that HWs' practices in relation to breastfeeding are influenced by a number of factors, not just exposure to training and information (Gouws et al., 2005). Designing an effective intervention to promote behaviour change, necessitated knowledge of the context in which change should occur, including the social and physical environment. This intervention formed part of a quasiexperimental study to assess the impact of this approach on attitudes and practices of front‐line HWs in South Africa. The aim of this paper is to describe the development and implementation of this intervention and assess if this model could be adapted to provide a generic tool for CME for HWs.

## 2 METHODS

### 2.1 Study setting

The intervention was conducted in primary health care clinics in two districts in South Africa. These were Tshwane (urban district in Gauteng Province) and Ugu (rural district in KwaZulu‐Natal Province). These districts were selected for their differing historical infant feeding contexts: KwaZulu‐Natal has a history of strong political will to support breastfeeding, whereas in Gauteng, women living with HIV historically practiced predominantly formula feeding. Further, antenatal HIV prevalence is high in both Ugu (45.9%) and in Tshwane (25.3%; South Africa Department of Health, 2017). Clinics in both areas provide a similar range of services to mothers and children, with several cadres of HWs in different clinical areas provide infant feeding counselling, including registered nurses, enrolled nurses, nutrition advisors, and HIV counsellors, working in the antenatal, postnatal, and child health sections of the clinic.

### 2.2 Theoretical basis for the intervention

**Figure 1:**
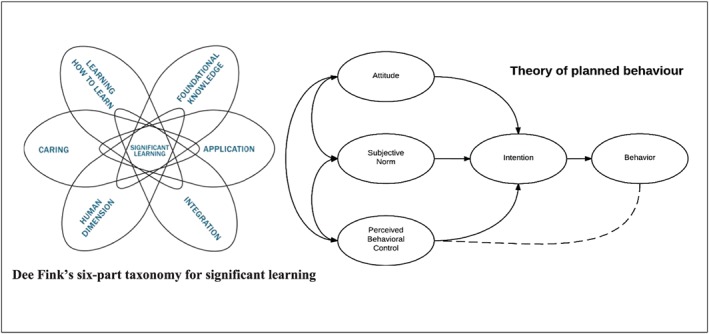
Theoretical frameworks that informed the development of the intervention

We used two theoretical frameworks to underpin the intervention. First, we used Dee Fink's six‐part taxonomy of significant learning (Figure 1). This suggests that for learning to be significant, it requires the development of a range of skills. In addition to developing foundational knowledge, learners should learn to apply skills, integrate ideas, develop new feelings/interests and values, as well as learning how to learn. This only occurs when a systematic approach is used to influence and change behaviours. For significant learning to occur, there needs to be a lasting and important change in the learner (Fink, 2013).

Second, we used the theory of planned behaviour (Figure 1; Ajzen, 1991). This theory suggests that an individual's intention to perform a behaviour is influenced by three constructs namely, (a) the person's attitudes towards performing the behaviour, (b) their beliefs about whether people who are important to them will approve of the behaviour (subjective norms), and (c) their beliefs about how likely they are to be able to implement the behaviour successfully.

Using these two theories, we compared the revised 2016 HIV and infant feeding guidelines with previous versions of the guideline to identify where changes to the knowledge, attitudes, and beliefs of HWs were required for them to implement the new guidelines. HWs providing infant feeding counselling in accordance with the revised guidelines needed to agree with the change, believe their colleagues and other stakeholders will approve of the action, and believe in their own ability to implement it successfully. Our intervention was developed to directly target HWs knowledge of HIV and infant feeding guidelines, to shift attitudes about the safety and importance of breastfeeding for HIV‐positive and HIV‐negative mothers, and to improve HWs confidence with breastfeeding counselling in the context of HIV. Other aspects targeted were to highlight the need for HWs to be open to learning through new approaches and to provide opportunities for HWs to practice integrating complex ideas such as ART adherence with viral load monitoring and breastfeeding support during the consultation.

### 2.3 Design of the intervention

In order to design the intervention, we first conducted a group discussion with a small group of HWs providing HIV and infant feeding counselling in one clinic, which we refer to as a “reference group.” Reference group participants included operational manager, registered nurses, enrolled nurses, and nutrition advisors. The aim of the discussion was to gain in‐depth understanding of the context where HIV and infant feeding guidelines were implemented, the challenges faced by front‐line HWs managing HIV‐infected mothers and their infants, and to identify gaps and barriers to implementation of the new guidelines. During the discussion, we explored current feeding practices among HIV‐infected mothers in the community and reasons for these feeding practices, as well as current infant feeding advice given in the clinic and the reasons for this advice. We explored knowledge gaps around HIV and infant feeding and discussed any common or difficult cases presenting at the health facility recently. In addition, we challenged the attitudes and beliefs of HWs about the safety of breastfeeding for HIV‐infected mothers. Finally, we introduced the new WHO guidelines and invited comparison with previous guidelines.

**Figure 2:**
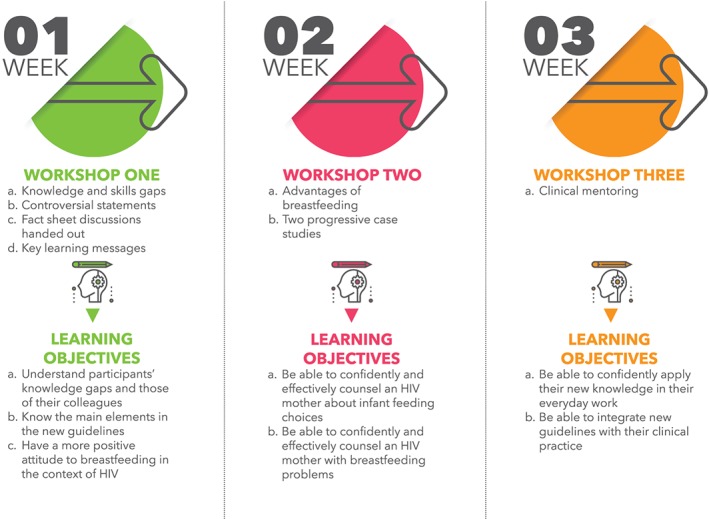
*Implementation activities and learning objectives*

On the basis of the findings of the reference group and inputs from a small group of senior researchers and clinicians experienced in the field of HIV and infant feeding, draft materials were developed. Learning objectives were outlined for each activity on the basis of the theories that underpinned the intervention. These were reviewed by the group of experts and adapted accordingly. Further, intervention activities were piloted in two clinics and adapted according to the experiences of participants and facilitators. Figure 2 outlines the activities and specific learning objectives for each session.

### 2.4 Intervention strategy

We used a participatory team‐based approach to promote team coherence and buy‐in from HWs within each health facility. The operational manager identified all HWs who provided nutritional counselling and support for infant feeding in each clinic, all of whom were invited to participate in the intervention. Participating HWs included the operational manager, professional and enrolled nurses, nutrition advisors, lay health counsellors, community HWs, and other visiting HWs such as dieticians, nutritionists, doctors, and clinic supervisors.

We chose to implement the intervention in the health facility, rather than off‐site, to provide context‐related mentoring and ensure that all cadres of HWs attended together. We aimed to build multidisciplinary, clinic‐based teams to facilitate peer learning and to clarify roles and responsibilities within the team. Clinic‐based learning also provided opportunities for individual clinical mentoring where facilitators directly observed the counselling skills of HWs and gave immediate feedback.

### 2.5 Structure of the intervention

The intervention was conducted in 12 clinics in each of two districts in two provinces of South Africa (KwaZulu‐Natal and Gauteng). Two facilitators, one from each district, delivered the mentoring workshops; both facilitators were experienced health professionals, one a registered nurse midwife and the other a nutritionist. To improve consistency across the two sites, facilitators worked together for the first two workshops, and model answers were developed, where appropriate, to guide the discussions. A senior team member provided additional support and accompanied facilitators to a number of workshops and mentoring visits in both sites.

Team workshops and clinical mentoring were provided over three sessions conducted in each participating clinic over three successive weeks. In order to minimise disruption to clinic services, the duration of the workshop was short (1–2 hr), and the scheduling was flexible and negotiated with the operational manager. Activities were designed to be conducted with few resources in a limited space; and therefore, a simple approach was taken using paper cards and pens, without slides, computers, or other technologies.

The first two workshops were group workshops, attended by the whole team (approximately 10 participants in each clinic); the third contact provided individual clinical mentoring to each participant. All sessions were conducted in the local language of the participants (either Setswana or isiZulu). At the end of each workshop, refreshments were provided. After completion of the series of workshops, we returned to the clinic to provide “catch‐up” workshops for participants who had been unable to attend one or more workshops to maximise participation.

After completion of the team workshops and clinical mentoring sessions, HWs were given access to project‐specific WhatsApp mobile phone groups, coordinated by their workshop facilitator. This aimed to provide a safe space for sharing concerns about clinical problems and counselling tips to support peer learning. In addition, key messages were posted on the WhatsApp group to serve as reminders of the knowledge gained. The WhatsApp group was supervised by the project team.

### 2.6 Workshop content and activities

During the first workshop, three activities were conducted, and HWs were provided with a fact sheet that included all important knowledge items. Ten key learning messages were identified by the study team, and these were highlighted as they arose during the workshops, and the messages were pinned to the wall. These messages remained in the health facility during the intervention period to serve as reminders of key messages about HIV and infant feeding.

At the start of the first team workshop in each facility, participants were asked to write all their *knowledge and skills gaps* relating to breastfeeding, particularly in the context of HIV, on small cards (Activity 1). These cards were pinned on the wall, and participants were then asked to attach a sticker to their three most important knowledge gaps to identify the main knowledge gaps for the group. All knowledge gaps were addressed over the three workshops.

In the second activity, participants were given a series of paired *controversial statements* (Box 1) and asked to identify which of the two statements more closely represented their own knowledge, beliefs, and attitudes about HIV and infant feeding. Statements were not intended to be correct or incorrect but aimed to stimulate discussion around difficult issues. Four paired controversial statements were used in this activity.

**Table:** 

Box 1 Controversial statements	
Statement	Statement
Benefits of breastfeeding outweighs the risk of acquiring HIV	Breastfeeding puts HIV exposed infants at risk
Mixed feeding before 6 months of age should be avoided for HIV positive mothers because it significantly increases the risk of HIV transmission	Although EBF is recommended, mixed feeding in the first 6 months of age is not a reason for HIV+ mothers on ART to stop breastfeeding
An HIV infected mother on ART who has missed eight doses of her treatment this month should have a viral load test done and stop breastfeeding immediately	An HIV infected mother on ART who has missed eight doses of her treatment this month should have a viral load done, restart ART and continue to breastfeed
I believe it is possible for HIV positive mothers who are away from their baby to express breastmilk for their baby to receive while they are away	I believe it is too difficult for HIV positive mothers to express breastmilk while they are at work or school, and they should rather stop breastfeeding to avoid mixed feeding

In the next workshop, HWs were asked to identify all the *advantages of breastfeeding* that they knew, write these on cards, and pin the cards to the wall. Any breastfeeding benefits not identified by participants were added by facilitators. To help HWs apply their knowledge about the advantages of breastfeeding more effectively and to explore their own attitudes, they were asked to identify the one advantage that would most effectively motivate or influence individuals with different characteristics to choose to breastfeed or to support breastfeeding. Different individuals given as examples included themselves or a member of their own family, teenage mothers, working mothers, older mothers, or grandmothers. In this way, HWs were encouraged to tailor their infant feeding messaging to the context of individual mothers.

To promote application of skills and integration of ideas, we developed progressive case studies (Box 2) to challenge HWs to think critically about the clinical decisions they make and the counselling they provide. These were discussed in the second workshop. The case studies were challenging cases about HIV‐positive breastfeeding mothers and included early introduction of food and fluids other than breastmilk, inconsistent ART adherence, high viral loads, and issues related to the need to return to work or school. This learning strategy promotes learning from shallow‐to‐deep or significant knowledge, combining knowledge, attitudes, and beliefs as described in the theoretical frameworks.

**Table:** 

Box 2 Example of progressive case study
1. A 16 year old girl comes to the clinic for a postnatal check at 6 days. She tells you she is breastfeeding and she is HIV positive. What questions do you need to ask? Is there anything you need to do for this patient? 2. Her breasts are very painful and engorged, and it is painful when she breastfeeds. The baby is crying a lot, is restless and wants to feed all the time. She is tearful and she wants to stop breastfeeding. How would you manage this information? Are there any further questions you would like to ask? Is there anything you need to do for this patient? 3. She tells you that she has been giving formula milk for the past two days as she is worried that the baby is hungry. How would you manage this information? Are there any further questions you would like to ask? Is there anything you need to do for this patient?

In the third contact session, individual clinical mentoring was provided during consultations conducted by HWs providing counselling to mothers attending the health facilities with young infants. If there were no suitable mothers available on the day, a simulated counselling session using role play was used and individualised feedback was provided to the HW.

### 2.7 Workshop evaluation

Workshop evaluations were undertaken following each team workshop, using a 4‐point Likert scale to assess participants' experiences of the workshop. Open‐ended questions were included to assess participants' perceptions about the workshops, their main learning points, and how they would change their clinical practice as a result of the workshop.

Data were entered into an Excel spreadsheet and converted to SPSS Statistics V25 for analysis. Simple frequencies were presented for quantitative data. Open‐ended questions were coded thematically, and the number of times a particular theme was mentioned was tallied. In total, 429 workshop evaluations were submitted: 228 from the first workshop and 201 from the second workshop. Questions were the same for both evaluations so to avoid repetition; data presented are from the second workshop only.

### 2.8 Ethical approval and consent to participate

Ethical approval was obtained from South African Medical Research Council (EC028‐9/2016) and the Biomedical Research Ethics Committee at the University of KwaZulu‐Natal (BREC: RECIP348/17), and permission for the study was obtained from Gauteng and KwaZulu‐Natal Departments of Health. All participants provided written informed consent.

## 3 RESULTS

### 3.1 Attendance at workshops

Overall, 303 HWs were identified by operational managers in 12 clinics to participate in the intervention. As planned, 48 mentoring workshops (two per participating clinic) and 24 1‐day clinical mentoring sessions (one per participating clinic) were conducted. Participation was assessed using workshop attendance registers. In most cases, there were some participants who were unable to attend all the scheduled workshops, and facilitators returned to the clinic for catch‐up workshops or clinical mentoring visits to improve coverage. Participation in team workshops, clinical mentoring sessions, and catch‐up sessions are shown in Table 1.

**Table 1:** Attendance at team workshops and clinical mentoring session

	Attended workshop (*n*)	Attended catch‐up (*n*)	Total attended, *n*/*N* (%)
Team workshop 1	202	63	265/303 (87.5)
Team workshop 2	223	34	257/303 (84.8)
Clinical mentoring 3	216	40	256/303 (84.5)

### 3.2 Knowledge gaps prioritised by HWs

ART adherence monitoring during breastfeeding and management of viral load results was the most common knowledge gap prioritised by 173/265 (65.3%) participants in Workshop one. Other important knowledge gaps included how to manage breast conditions including cracked and/or bleeding nipples (79/265; 29.8%); when to advise mothers to stop breastfeeding (68/265; 25.7%); how to advise mothers to express and store breastmilk (64/265; 24.1%); when it is safe to breastfeed (32/265; 12.1%); how to advise mothers who want to formula feed or who are mixed feeding (14/265; 5.3%); and how to counsel HIV‐discordant couples about breastfeeding (12/265; 4.5%).

### 3.3 Workshop evaluation

HW responses to the workshops were positive and indicated they would change the advice they give to HIV‐positive mothers as a result of the workshop (Table 2).

**Table 2:** Responses to workshop evaluation

Questions asked	Agreed or strongly agreed (*N* = 201)	Percentage
Workshop format		
I enjoyed the way the workshop was delivered (format of the workshop)	198	98.5
I would have preferred to have a lecture with the information that I need to know	111	56.3
The workshop was too long	37	18.6
Workshop content		
The workshop provided me with information that I did not know previously	179	89.1
The workshop did not provide any new information	30	15.2
The effect of the workshop on me		
Because of the workshop, I will change the advice I give HIV positive mothers about breastfeeding	198	98.5
I feel confident to practice what I learnt in this workshop	192	95.5

### 3.4 Key learnings

A total of 201/257 participants in Workshop 2 completed an evaluation. Participants were asked to write down their most important learnings. The following topics were recorded by participants: benefits and importance of breastfeeding (68/201), management of breastfeeding challenges (26/201), adherence to ART (25/201), how to express and store breastmilk (18/201), HIV and prevention of mother‐to‐child HIV transmission (15/201), management of mixed feeding (10/201), and viral load monitoring (9/201).

When asked about how they would change their practice to align with the knowledge and skills gained from attending the workshops, participants most often said they would change their messages about advantages/benefits of breastfeeding (66/201); and importance of breastfeeding, exclusive breastfeeding, and continued breastfeeding for 2 years (43/201). A smaller number of HWs suggested that they would change the advice they would give mothers who complained of breast engorgement and cracked nipples and expressing and storing breastmilk for mothers who returned to work or school.

### 3.5 Facilitators' experiences during implementation of the workshops

There were numerous challenges during implementation of the intervention. Despite arranging the workshops for a time that suited participants, workshops frequently started late and participants had to be called from their workplace by facilitators. Operational managers reported that the workshops were too long. As a result, activities were rushed or had to be moved to the next workshop. Workshops were often disrupted while participants moved in and out to attend to patients, and many participants were not able to stay for the full length of each workshop. Because workshops were conducted in the clinic, space and resources were limited, and workshops were conducted in small and sometimes inappropriate settings, like the kitchen, making full participation in workshop activities challenging.

Using WhatsApp groups to provide peer support was also a challenge and was not well utilised by participants, and those case studies that were posted by HWs were difficult and often required high‐level input from outside the WhatsApp group. As a result, HWs had to be contacted directly rather than via the group due to the complexity of support required and issues of confidentiality, thus defeating the intention of providing peer support.

## 4 DISCUSSION

We developed and tested a novel mentoring intervention to improve dissemination of new HIV and infant feeding guidelines, which was (a) based on widely applicable educational principles; (b) a flexible, team‐based approach that employed peer‐to‐peer learning driven by the participants' knowledge gaps; (c) aimed at low‐resource settings; and (d) conducted in the work place using simple, low‐cost learning techniques that have shown to be easily replicated and implemented at scale. In the evaluations, participants reported that all identified knowledge gaps were addressed and that their newly acquired knowledge, skills, and confidence would lead to changes in practices when managing HIV‐infected breastfeeding women. However, although most HWs reported enjoying the training format, some still stated that they would have preferred a lecture. The reasons for this is unclear but may be because our approach was perceived as more time consuming. However, didactic methods have consistently shown to be ineffective (Bluestone et al., 2013).

We applied educational principles and a theoretical framework to guide development of the workshop content (Ajzen, 1991; Fink, 2013) informed by the reference group, who provided crucial insights into the issues facing HWs on the ground. The reference group was a novel approach that strengthened our ability to ensure the training was informed by HWs experiences and concerns. Thus, we ensured that learning activities were appropriate to the context, current knowledge, and clinical role of participants and built on participants' existing knowledge. This contrasts with many training courses that are developed by experts in isolation, on the basis of assumptions about the context and learning needs of potential participants, and fails to take account of or build on participants' existing knowledge and attitudes. Previous studies have shown the importance of embedding skills development approaches in participants' context (Poot et al., 2018; Siron et al., 2015).

Further, we chose to conduct the workshops on‐site to promote attendance and minimise service disruption but more importantly, we intended that the workshop content be strongly rooted in the work environment (Siron et al., 2015) so that groups of HWs working together on a daily basis could learn together and implement the new guidelines with team members fulfilling different roles according to their scope of practice. We think that this approach, although challenging in terms of the learning environment and competing clinical responsibilities, was nevertheless valuable. On‐site learning should be contrasted with the alternative of bringing HWs from several clinics together for a workshop away from the work setting, when many HWs in each clinic, usually those from the lower cadres, are excluded and have to rely on feedback being provided (Mbonye et al., 2016). This may actively undermine the adoption of new guidelines and practices if some staff are out of date and continue with outdated practices; it is likely that over time, the change will be lost and clinical practices return to the status quo. Off‐site training may also be costly requiring a venue, catering, and transport for participants, whereas on‐site mentoring or training can be implemented within routine clinic visits by clinic supervisors or primary health care trainers. Trainers and supervisors have a defined and established role in the clinics and may be able to ensure HW participation more easily than our project‐based team. However, given the challenges experienced, we would suggest that clinic teams attend in smaller groups to reduce pressure on clinical services. For example, workshops could be conducted twice in a single day, or on two separate occasions, reducing the need for catch‐up sessions. In addition, structured activities with clear guidance on how activities and discussions should be conducted mean that minimal facilitation skills or training of trainers would be required.

In addition, it is important to focus learning towards changing practices rather than only on acquiring new knowledge. Fink's (2013) taxonomy of significant learning informed the development of learning activities to ensure that significant and sustained learning was achieved. This approach suggests that although foundational knowledge is important, it is insufficient to lead to lasting change and learners must be able to apply and integrate new knowledge. We focussed learning activities on supporting learners to apply their new knowledge and integrate it with their existing practice using a variety of exercises to challenge current practices and promote active discussion about the changes required. The taxonomy also suggests that the human dimension of learning be addressed if significant learning is to be achieved, acknowledging that learning about how learners and others act in response to a situation, helps learners interact more effectively. In this case, HIV has societal and cultural dimensions in addition to being a medical condition, so it is important for participants to understand their own behaviour and attitudes as well as those of their patients (Piwoz et al., 2006). Controversial statements were a simple and successful learning tool that served to challenge the beliefs and attitudes of HWs while improving their practical knowledge through team‐based peer learning.

The theory of planned behaviour suggests that for HWs to successfully change their practices, they need to have a positive attitude to the proposed change, believe that they will be able to implement the change, and that their peers and other stakeholders will support them. Peer‐to‐peer learning with senior clinic managers and lower HW cadres attending the same workshops ensures that team members learn together and develop a common vision and has been successfully used among teams of medical students (Sin et al., 2019) and primary care practitioners (Coleman et al., 2019). Where team members have different roles and scope of practice, implementation can be coordinated between different members of the team. Practical exercises, case studies, and clinical mentoring were used to improve self‐efficacy and ensure that HWs developed practical hands‐on skills to improve their confidence in managing difficult and complex clinical problems of HIV infected mothers. However, it should be recognised that this approach requires considerable skills and resource to develop the training package.

We used mobile messaging to provide ongoing support following the completion of the workshops. This approach has been successfully used in South Africa with pregnant women and is currently being formally evaluated (Zunza, Cotton, Mbuagbaw, Lester, & Thabane, 2017). It has also been used with patients in other settings and with different conditions; in particular, this approach has been shown to improve ART adherence (Mbuagbaw et al., 2013). Although there is little experience of using this approach with HWs, it seemed likely that a WhatsApp group could provide opportunities for peer‐to‐peer leaning and support. However, this proved challenging because individual cases posted were complex and did not lend themselves to being discussed in a public forum. Key messages were included on the WhatsApp group to reinforce those given in the workshops, but it was unclear how well these were received. Further piloting and evaluation are required to develop effective cell phone messaging with HWs especially as other similar initiatives to support front‐line HWs in South Africa, such as NurseConnect, are currently being implemented (South Africa Department of Health, 2019).

## 5 CONCLUSION

We believe that this innovative, team‐based, participatory mentorship intervention for disseminating clinical updates on‐site provides a workable, sustainable approach to updating HWs in resource‐constrained settings, which can be adapted to other clinical topics. This is a departure from conventional didactic approaches because it is embedded in the context where skills are needed, conducted with the team working together, and driven by knowledge gaps of participants. Despite the challenges of providing on‐site training and mentoring, we believe the use of existing clinic supervisors and trainers would make this a sustainable approach. Further research and evaluation is required to understand these issues better; in particular, qualitative methodologies would have added to the understanding of how HWs experienced the training, and further evaluation is required to determine effectiveness at scale.

## CONFLICTS OF INTEREST

The authors declare that they have no conflicts of interest. The findings and conclusions in this paper are those of the authors and do not necessarily represent the official position of WHO.

## CONTRIBUTIONS

CH, AG, TD, LH, UF, VJ, NR, MK, and DS participated in the conceptualisation of the intervention and/or design of the study. CH, LH, AG, TD, IE, and TT participated in the analyses and interpretation. All authors were involved in reviewing and contributing to the manuscript and approved the final version for submission.
